# Using systems thinking to study the coordination of the water–sediment–electricity coupling system: a case study on the Yellow River

**DOI:** 10.1038/s41598-021-01578-8

**Published:** 2021-11-09

**Authors:** Yuansheng Zhang, Zhiwei Cao, Wei Wang, Xin Jin

**Affiliations:** 1 Tianjin University, Tianjin, China; 2 Key Laboratory of Water Management and Water Security for Yellow River Basin of Ministry of Water Resources (Under Construction), Yellow River Engineering Consulting Co. Ltd, Zhengzhou, China

**Keywords:** Hydrology, Engineering

## Abstract

Joint operation of the Longyangxia and Liujiaxia reservoirs (Long-Liu operation) is of great significance for water and sediment regulation in the Yellow River. The water–sediment–electricity coupling system is a giant system with complex nonlinear relationships. A reliable Long-Liu operation scheme facilitates maximization of the benefits of the water–sediment–electricity system. Based on systems thinking, this paper quantitatively evaluated the reliability of different Long-Liu operation schemes and coordination of the water–sediment–electricity coupling system through the entropy weight method and dissipative structure model. The results indicated that the current operation scheme is more reliable than the adjusted scheme at the inter-annual scale and during the summer-autumn flood season and ice flood season within a year. However, the operation scheme should be improved during the spring irrigation period. The key factors influencing the quality of the water–sediment–electricity system include the outflow of the Liujiaxia reservoir, incoming sediment load into the Yellow River at Toudaoguai, sediment inflow-outflow difference in the Ningxia-Inner Mongolia Reach, water flow at Lanzhou and power generation upstream of Toudaoguai. The water–sediment–electricity system under the current Long-Liu operation scheme is more coordinated than that in the adjusted state, but the overall coordinated development of the system remains at a low activity level.

The Yellow River is a vital lifeline providing water resources in northern China, which functions as a life-supporting system for the survival and development of humans. In the Yellow River basin, problems have frequently occurred, such as shortages, uneven spatiotemporal distribution of water resources, and uncoordinated relationship between water and sediment^1^. As a living body, the Yellow River exhibits its own life attributes, and maintenance of its health is the ultimate goal of harnessing the Yellow River, which is embodied in four important aspects, including "no breaching of the embankment, no zero flow in the river channel, no pollution exceeding standards, no riverbed rise"^2^. Since the founding of the People's Republic of China, a series of water conservancy and hydropower works has been built or planned along the Yellow River, e.g., Longyangxia, Liujiaxia and Xiaolangdi. Joint operation of the reservoir group plays an increasingly important role in flood control and disaster alleviation, water resources regulation, and improvement of the relationship between water and sediment^3–5^. The reservoir group operation system involves water, sediment and power generation targets, and various complex relationships occur between natural and human factors. The operation process is carried out on a multilevel spatial scale, with the participation of many departments such as basin institutions, hydropower complexes, power grids and water receiving departments, thus forming a giant complex system^1^. In this giant system, the operation process must achieve tradeoffs in terms of water, sediment and electricity because contradictions exist considering the corresponding water requirements. In addition, water, sediment and electricity interact and influence each other through complex and non-linear relationships and feedbacks. Systems thinking can be considered a useful tool to better understand the various processes and interrelationships within a complex system, to effectively support the decision-making process^6^. Therefore, investigating this complex water–sediment–electricity giant system based on systems thinking is of great practical value for regional water resources management and river health.

Optimal joint reservoir group operation can fully manifest the hydrological and storage capacity compensation effects between reservoirs, maximize the utilization rate of water resources and optimize the comprehensive benefits^7^. Traditional optimal reservoir operation first obtains a feasible operation decision-making solution set by setting multiple targets and constraints^8,9^. Then, the optimal operation scheme is searched with algorithms, which have been developed from traditional dynamic programming algorithms, such as linear programming (LP), nonlinear programming, mixed-integer linear programming and fuzzy models^10–15^ into intelligent optimization algorithms, such as neural networks, genetic algorithms, partical swarm optimization and differential evolution algorithms^16–24^. In operation research, it is crucial to select the appropriate evaluation system based on the solution set of feasible operation decision-making schemes and to optimize the decision-making scheme that can maximize benefits according to the evaluation model of utility theory. As the cascade water, sediment and electricity joint operation system in a given basin is a giant complex system with multiple factors, levels, objectives, constraints, functions and stages^1^, comprehensive integration methods combining qualitative and quantitative approaches have thus been widely applied in operation scheme evaluation, such as the analytic hierarchy process, comprehensive index evaluation method, TOPSIS, gray correlation method, and improved entropy method^25–28^. Based on information theory, the entropy method determines the index weight according to the amount of utility information contained in data and then comprehensively evaluates the research object. This method can avoid subjective interference, thus achieving greater credibility^29^. Li et al.^27^ applied the analytic hierarchy process and entropy weight method to determine the weight coefficient of evaluation indices and then performed grey correlation analysis to rank several design schemes to accomplish scheme optimization. Because multiple evaluation indices at different levels can be considered in the entropy weight method, this technique can overcome the incompleteness of considering problems with a single index in terms of objectivity, and this method has been adopted to determine the operation gain distribution in cascade hydropower stations and obtain fair and reasonable allocation results^30–32^. In the application of decision-making evaluation, the lower the decision-making uncertainty and the higher the reliability are the lower the entropy and the higher the decision-making level^33^. In addition, compared to other methods, with the variation in each index value, the information entropy and weight in the system will change accordingly, and reservoir operation schemes can be better evaluated at the different stages.

In the Yellow River, the relationship between water and sediment is the key factor of river evolution. Currently, the flood risk remains the greatest threat, and the conditions required to ensure water resources are poor in the Yellow River. In terms of the operation object, integrated joint operation in the mainstream of the Yellow River combines three main goals, i.e., water volume, sediment and power generation, of which water and electricity comprise the lifeblood of the national economy, and sediment affects the health conditions of the hydropower complex and river. Water, sediment and electricity are closely interrelated. They interact and influence each other but also enable coordinated development. In the operation process, the determination of electricity with water is one of the important principles. Moreover, choosing water as the core, the complex correlation relationship between sediment and electricity can be established^1^. In the water–sediment–electricity coupling system, operation decisions should be scientific and reliable, which is beneficial to improve coordination among water, sediment and electricity, thus ensuring the evolution of these three aspects along a more coordinated direction. Higher coordination of the coupling system could benefit river safety maintenance and could support social-economic development. As a new stage in the development of system science, dissipative structure theory can be considered to evaluate the evolution of complex systems. The Brusselator model is a mathematical model to quantitative analyze dissipative structures, and Zhao et al.^34^ applied this model to study the evolution of a complex water resources system and the influence of internal and external factors on the system. However, currently, there are few reports on the application of this model in the assessment of operation schemes by evaluating the coordination of the water–sediment–electricity system under the influence of these reservoir operation schemes.

The Longyangxia and Liujiaxia reservoirs located in the upper reaches of the Yellow River, possess the capacity to enable multiyear regulation and incomplete annual regulation, respectively, and these reservoirs facilitate flood control, ice prevention, water supply, irrigation, etc. Joint operation of the Longyangxia and Liujiaxia reservoirs (Long-Liu operation) can regulate the multiyear runoff of the Yellow River, improve the guaranteed output and power generation of upstream cascade hydropower stations, distribute the water resources of the whole river and regulate the relationship between water and sediment. It is highly important to ensure the safety of water use in this river basin^3,22,23,35^. In this paper, the Longyangxia and Liujiaxia reservoirs and the upper reaches of the Yellow River directly affected by these two reservoirs were selected, and the uncertainty and reliability of operation decisions at different time scales were analyzed with the theoretical concepts of information entropy and dissipative structure. Moreover, the coevolution trend of the water–sediment–electricity system was assessed to establish a new evaluation framework for joint operation of reservoirs (group), thereby providing scientific support for management decisions and operation of the Yellow River under changing conditions.

## Study area

### Joint reservoir operation

**Figure 1:**
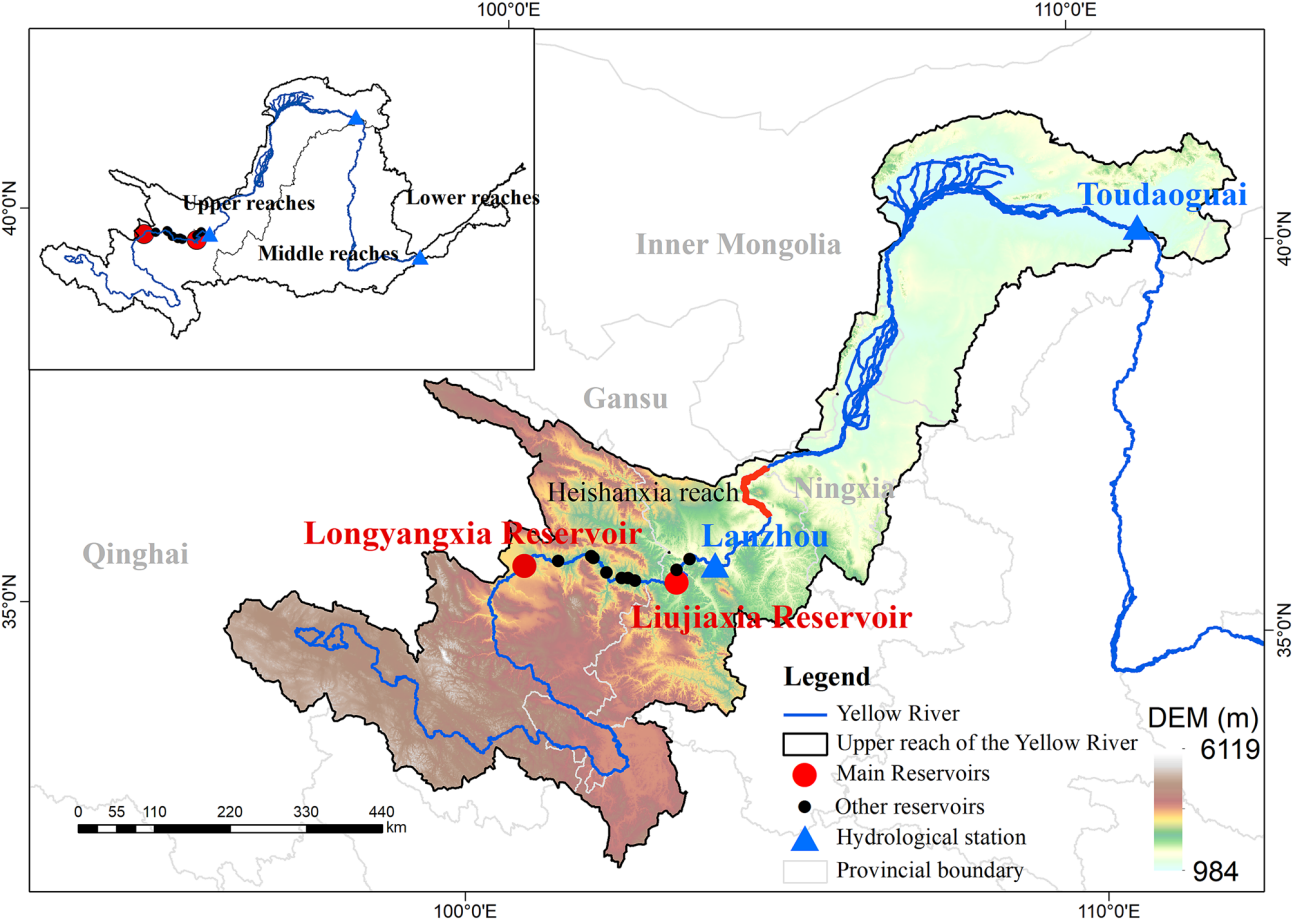
Schematic diagram of the locations of the Longyangxia and Liujiaxia reservoirs and the upper reaches of the Yellow River.

The Longyangxia Reservoir, located at the entrance of the canyon at the junction of Gonghe County and Guinan County, Qinghai Province, in the upper reaches of the Yellow River, is a large-scale comprehensive water conservancy project with a multiyear regulation performance. This reservoir controls an area of 131,420 km^2^ (17.5% of the Yellow River basin) and more than 1/3 (35.3%) of the runoff (205 × 10^8^ m^3^) of the whole river. Based on flow records from July 1989 to June 2016, the mean annual inflow and sediment load are 185 × 10^8^ m^3^ and 2490 × 10^4^ t, respectively. The Longyangxia Reservoir has a regulated storage capacity of 193.5 × 10^8^ m^3^. The storage coefficient, reflecting the regulation ability of the reservoir and defined as a ratio of regulated storage capacity to mean annual inflow of the reservoir, is 0.94. Thus, it is a multiyear regulation reservoir with regulation period longer than 2 years. It could also accomplish regulation at the daily, weekly and annual scale. The Longyangxia Reservoir focuses on power generation and comprehensively considers flood control and water supply utilization. The Liujiaxia Reservoir is located in the mainstream of the Yellow River in Yongjing County, Gansu Province, 100 km away from Lanzhou city (Fig. 1). This reservoir exhibits a mean annual inflow and mean sediment load (1989.7–2010.6) of 220 × 10^8^ m^3^ and 8700 × 10^4^ t, respectively. The normal impounded level reaches 1735 m. and the regulated storage capacity is 35 × 10^8^ m^3^. The regulation storage coefficient of Liujiaxia Reservoir is 0.16. Thus, it is an incomplete annual regulation reservoir. It exhibits a certain regulation storage capacity and can store a portion of the runoff during water-rich periods and release water during dry seasons or in dry years. The Liujiaxia Reservoir focus of this reservoir includes power generation and flood control, and comprehensive water supply and irrigation utilization are considered.

Joint operation of the Longyangxia and Liujiaxia reservoirs is expected to control flooding in the upper reaches of the Yellow River and can further mitigate the risk of ice floods, which refers to the swelling of rivers due to melting ice and snow in the upper reaches in the absence of thawing in the lower reaches. In addition, joint operation could help to satisfy the industrial and agricultural water needs in Qinghai, Gansu, Ningxia and Inner Mongolia and could improve the water supply during dry years in the middle and lower reaches of the Yellow River and the efficiency of upstream cascade power generation.

#### Index system

According to *Measures for Dispatch Management of Flood Control (Ice Prevention) of Reservoirs and Hydropower Stations in the Main Stream and Important Tributaries of the Yellow River (Trial)* (HFZB [2014] No. 34), it has been determined that the dispatch of flood control (ice prevention) measures in the Yellow River should follow the principle of electricity generation after water supply distribution to realize integrated water, sediment and electricity utilization and maximize the comprehensive benefits. In this paper, an evaluation system for the Long-Liu operation scheme is constructed considering reservoir operation and the characteristics of water and sediment in the lower reaches of the above reservoirs following the principles of standardization, comprehensiveness and accessibility.

**Figure 2:**
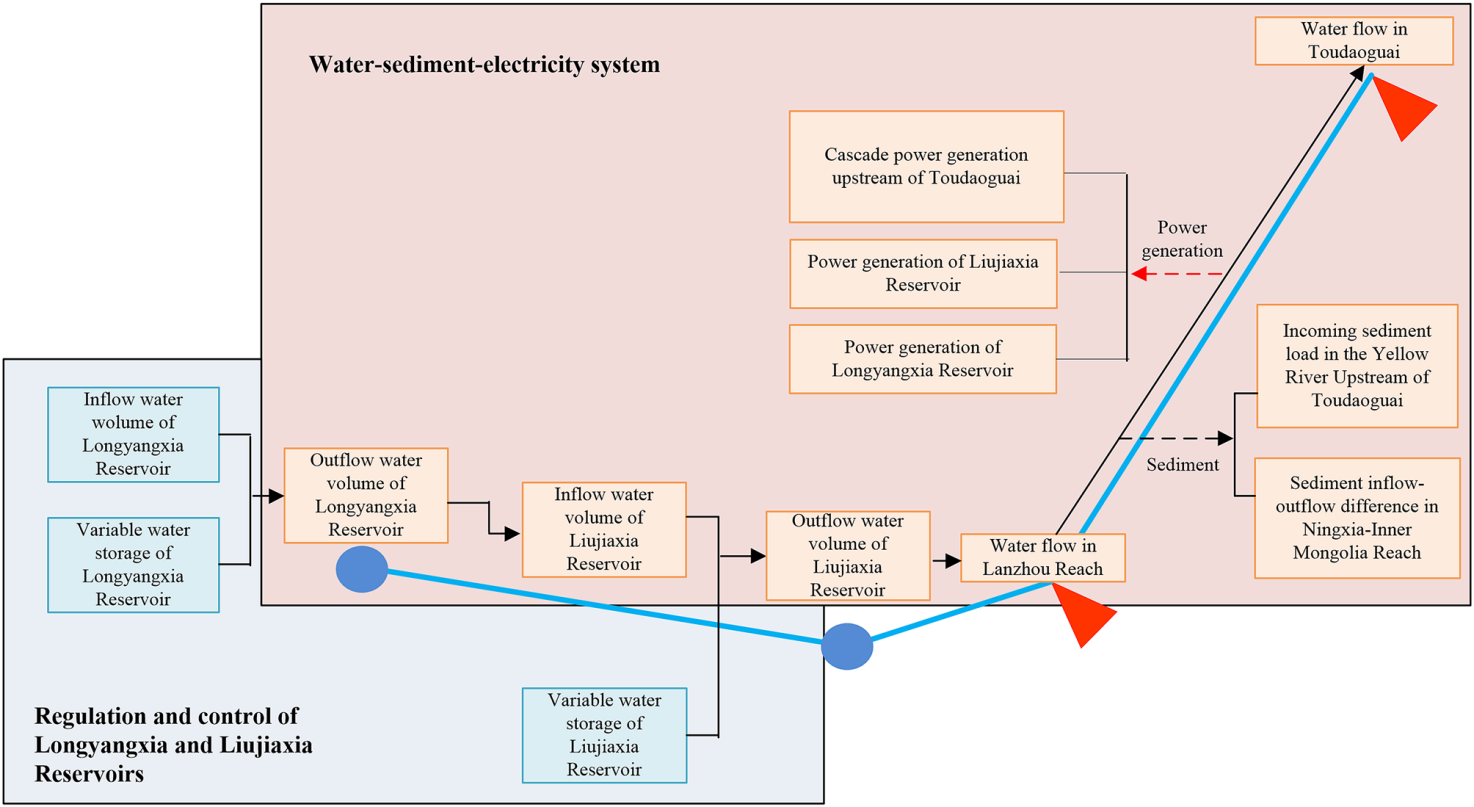
Schematic index classification of the operation system of the Longyangxia and Liujiaxia reservoirs.

The established evaluation index system is divided into three levels: target level, criterion level and index level. The target level is divided into the water–sediment–electricity system and operation control system of the Longyangxia and Liujiaxia reservoirs. During the development of the water–sediment–electricity system, the upstream reservoir group continuously provides the system with material, energy and information through joint operation. Therefore, in this paper, the water–sediment–electricity system is regarded as the internal system and the operation control system of the Longyangxia and Liujiaxia reservoirs is considered the external system affecting the development of the system. The criterion level includes operation control, reservoir flood control benefits, sediment discharge and siltation reduction benefits and power generation benefits, of which the latter three belong to the water–sediment–electricity system. The third level is the index level, which is described in Table 1 and Fig. 2.

**Table 1:** Construction of evaluation index system.

S. no.	Target level	Criterion level	Description of index	Unit	Attribute of index
1	Operation control system	Operation control	Inflow water volume of Longyangxia Reservoir	10^8^ m^3^	Negative entropy flow
2			Variable water storage of Longyangxia Reservoir	10^8^ m^3^	Negative entropy flow
3			Variable water storage of Liujiaxia Reservoir	10^8^ m^3^	Negative entropy flow
4	Water–sediment–electricity system	Flood control benefit	Outflow water volume of Longyangxia Reservoir	10^8^ m^3^	Positive entropy flow
5			Inflow water volume of Liujiaxia Reservoir	10^8^ m^3^	Positive entropy flow
6			Outflow water volume of Liujiaxia Reservoir	10^8^ m^3^	Positive entropy flow
7			Water flow at Lanzhou	m^3^/s	Positive entropy flow
8			Water flow at Toudaoguai	m^3^/s	Positive entropy flow
9		Sediment discharge and siltation reduction effect	Incoming sediment load in the Yellow River upstream of Toudaoguai	10^8^ t	Positive entropy flow
10			Sediment inflow-outflow difference in Ningxia-Inner Mongolia Section	10^8^ t	Positive entropy flow
11		Power generation effect	Power generation of Longyangxia Reservoir	10^8^ kW h	Positive entropy flow
12			Power generation of Liujiaxia Reservoir	10^8^ kW h	Positive entropy flow
13			Cascade power generation upstream of Toudaoguai	10^8^ kW h	Positive entropy flow

## Data and methods

### Data

The time span of the collected index data ranges from July 1956 to June 2010. The hydrological year is adopted as the annual measurement scale in monthly time steps. In this study, two scenarios, including the current Long-Liu operation scheme and the pre-Long-Liu operation scheme, were set. Indicator values under the above two scenarios were calculated with the Yellow River Simulation Model (YRSIM)^36^. The model input data included three aspects: (1) long-term runoff records of hydrological stations at the monthly scale pertaining to the main stream of the Yellow River, (2) industrial, agricultural, ecological and domestic water demands in counties in the Yellow River basin, and (3) reservoir characteristics, such as storage-capacity curves, stage-discharge relationships, evaporation and leakage loss coefficients and discharge limits. Under the pre-Long-Liu operation scenario, the regulation storage capacities of Longyangxia and Liujiaxia reservoirs were set to zero, suggesting that runoff naturally occurs without disturbance by any reservoir operation. The other conditions were kept the same as those under the current Long-Liu operation scenario in the simulations.

### Methods

In this study, the water–sediment–electricity coupling system is a giant complex system with characteristics of openness, principal equilibrium, system fluctuation and internal nonlinear action, so this system possesses the conditions to establish a dissipative structure^34^. The system continuously evolves under the influences of both internal factors (i.e., river channel inflow, incoming sediment load, and sediment inflow-outflow difference in the reach) and external factors (i.e., operation and regulation of the reservoir group). During system evolution, the internal positive entropy flow is the main cause of system disorder. With increasing positive entropy, the internal system disorder deepens. In contrast, external reservoir regulation could provide the internal system with material and facilitate information exchange. This process is referred as the introduction of negative entropy flow into the system, which could help to decrease the positive entropy and promote the system to achieve a new beneficial development mode (Table 1 and Fig. 2).

In this study, the uncertainty in the reservoir operation scheme was first evaluated with an entropy weight model. Then, the coordinated development level of the water–sediment–electricity complex system was investigated with the Brusselator model, which is a method to analyze dissipative structures and explore system development modes.

1. Entropy weight analysis

Values of the entropy (*S*) and entropy weight (*ω*) of each indicator were calculated according to Zhang and Vijay^33^ as follows:

1 $$S = - \frac{1}{\ln n}\sum\limits_{k = 1}^{n} {(p_{k} \ln p_{k} )}$$

2 $$p_{k} = \frac{{f^{k} }}{{\sum\limits_{i = 1}^{n} {f^{i} } }}$$

where, *p*^*k*^ is the proportion of the standard probability function value among all values *f*^*k*^.

3 $$A = \sum\limits_{i = 1}^{N} {(S_{i,A} \times \varpi_{i,A} )}$$

4 $$B = \sum\limits_{i = 1}^{N} {(S_{i,B} \times \varpi_{i,B} )}$$

5 $$\varpi_{i} = \frac{{1 - S_{i} }}{{N - \sum\limits_{i = 1}^{N} {S_{i} } }}$$

where ***N*** is the number of indicators, and *A* and *B* are the positive and negative entropy sums, respectively. The sum of *A* and *B* is the total entropy of the system. The lower the total entropy is, the lower decision uncertainty and the higher the decision quality.

In regard to each indicator, the smaller the *S*_*i*_ value is, the lower the uncertainty in the indicator, thus the larger the *ω*_*i*_ value, and the greater the contribution to the system. In each year, the sum of the weights of all indicators is 1, but the *ω*_*i*_ value of the indicators vary with *S*_*i*_, suggesting that the impact of each index on the system changes over time.

2. Dissipative structure analysis

In the evolution process of the water–sediment–electricity coupling system under the joint effects of internal and external factors, the entropy flow relationship of the system can be described with the Brusselator model according to Zhao et al.^34^ as:

6 $$\begin{aligned} & A\mathop{\longrightarrow}\limits^{{K_{1} }}X \\ & B + X\mathop{\longrightarrow}\limits^{{K_{2} }}Y + D \\ & Y + 2X\mathop{\longrightarrow}\limits^{{K_{3} }}3X \\ & X\mathop{\longrightarrow}\limits^{{K_{4} }}E \\ \end{aligned}$$

where model parameter *A* denotes the positive entropy of the water–sediment–electricity system, *B* denotes the negative entropy of the water–sediment–electricity system (i.e., the negative entropy introduced by the reservoir operation and control system), *X* and *Y* denote the quantifiable indices of the positive and negative entropy flows, respectively, into the system, and *D* denotes the lower coordinated development activity state of the system. In this state, the coordination effect is relatively poor, the system uncertainty is higher, the disaster risk is therefore higher and the comprehensive benefits of operation are lower. *E* denotes the higher coordinated development activity state of the system. In this state, the coordination effect is relatively good, the system uncertainty is lower, the disaster risk is thus lower and the comprehensive operation effect is higher.

In this study, the coordinated development activity level was assessed with dissipative structure index (*Index*_*DS*_). According to the equation and inference of the Brusselator model, the system exhibits a dissipative structure when the critical dynamics condition (i.e. $\left| B \right| > 1 - A^{2}$) is reached. Therefore, *Index*_*DS*_ is defined as:

7 $$Index_{DS} = \left| B \right| - (1 + A^{2} )$$

*Index*_*DS*_ < 0 and *Index*_*DS*_ > 0 indicate low and high coordinated development activity levels, respectively, of the system. *Index*_*DS*_ = 0 indicates the critical state of system “fluctuation” accumulation and activity change.

## Results

### Reliability of operation decisions

According to the monthly data, the negative entropy in the external operation control system and the positive entropy in the system are calculated at the interannual and monthly scales, respectively, and the sum of these entropy items is the total entropy. A lower negative entropy, i.e., a higher absolute value, indicates that external operation control promotes a more coordinated evolution of the water–sediment–electricity system, thus reducing the disorder attributed to internal system development, and the entropy reduction effect is more obvious.

**Figure 3:**
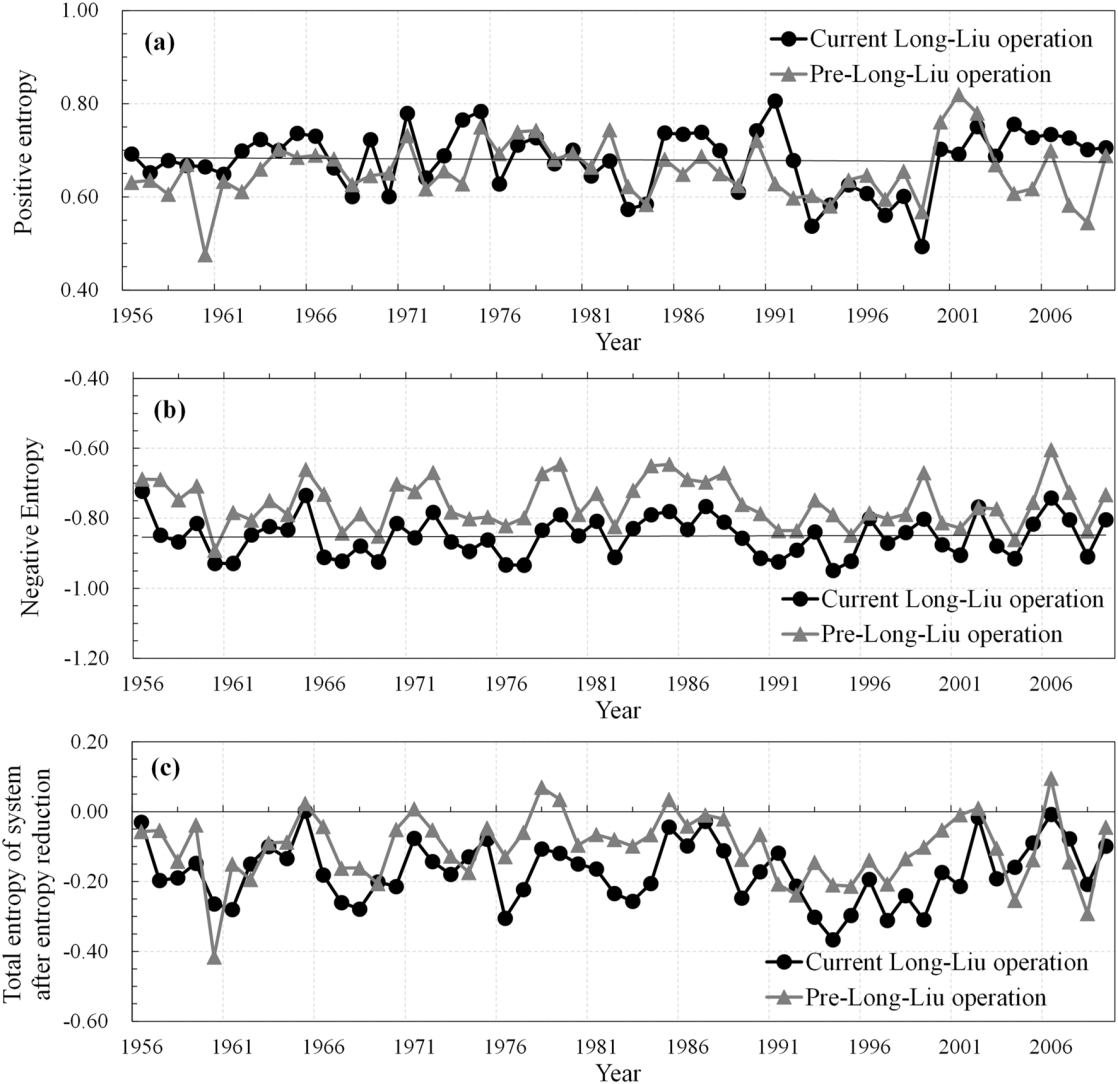
Comparison of the positive, negative and total entropy levels in the current situation and pre-Long-Liu operation state at the inter-annual scale.

At the interannual scale, the absolute value of the negative entropy in the current situation is approximately 0.85, which is approximately 12.4% higher than that (0.76) in the pre-Long-Liu operation state, and the interannual variation coefficients of the negative entropy in the current and pre-Long-Liu operation states are 0.57 and 0.66 respectively, indicating that the current Long-Liu operation scheme introduces a higher and more stable negative entropy flow into the water–sediment–electricity system (Fig. 3). In the current situation, the total entropy in the system after negative entropy reduction is 69.3% lower than that in the pre-Long-Liu operation state, indicating that the current Long-Liu operation scheme achieves a higher reliability and thus a higher system development quality (Fig. 3c).

According to the characteristics of the incoming water and water usage, the year can be divided into different periods, namely, the summer-autumn flood season (July–September), transition period (October), ice flood season (November–March of the following year) and water-consuming peak period (April–June)^37^.

**Figure 4:**
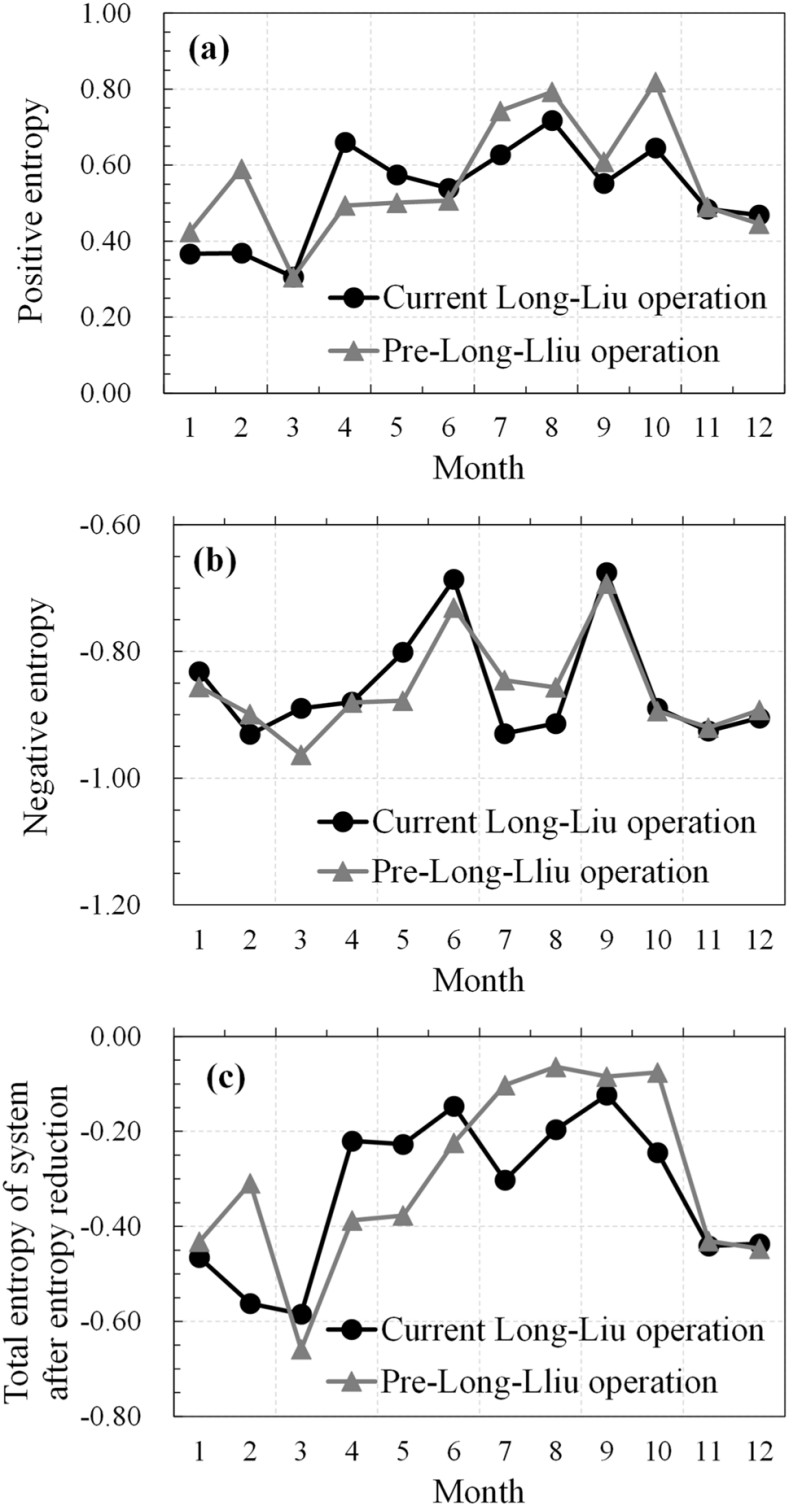
Comparison of the positive, negative and total entropy levels in the current situation and pre-Long-Liu operation state in the different months throughout the year.

The negative entropy values in both the current and pre-Long-Liu operation states are relatively low from July to August and from October to April of the following year. During the summer flood season from July to August, and the ice flood season from December to February of the following year, the negative entropy value in the current situation is lower than that in the pre-Long-Liu operation state, especially duting the autumn flood season (Fig. 4).

From July to September, the Longyangxia and Liujiaxia reservoirs regulate the water volume, thus greatly improving the flood control standards of the reservoirs downstream of Longyangxia and ensuring the safety of the upstream areas and even the whole river during the flood season^8,9^. From January to February, the Ningxia-Inner Mongolia reach occurs in the stable freezing period. Joint Long-Liu operation is conducive to controlling the stability of the discharge flow and maintaining stable operation of the Northwest Power Grid during the dry season. During these two periods, in the current operation situation, the reservoir introduces negative entropy flow into the system through scientific regulation and control, which effectively reduces the total entropy in the system, thus lowering the total entropy. However, from March to June, especially from April to June when the irrigation water consumption level in the Ningxia-Inner Mongolia irrigation area is higher, the total entropy in the current situation is higher than that in the pre-Long-Liu operation state (Fig. 4c), indicating that the current Long-Liu operation scheme attains a greater uncertainty and lower reliability during this period, and this scheme should be further optimized.

### Identification of the key (high weight) indicators and analysis of the contribution degree

The entropy weight of three external operation control indicators and ten internal effect indicators at the interannual scale during the study period (1956–2009) in the current situation and pre-Long-Liu operation state was calculated to analyze the contribution degree of these indicators and identify the key indicators. At the interannual scale, the most important external operation control indicators transitioned from the inflow water volume into the Longyangxia Reservoir (a probability of 74.1%) to the variable water storage capacity of the Liujiaxia Reservoir (a probability of 83.3%) in the current situation, highlighting the vital role of the Liujiaxia Reservoir in controlling flooding, reducing sediment discharge and siltation and power generation regulation under the current operation scheme. In terms of the internal system, the top five indicators based on the contribution degree attained a more uniform distribution across the three criterion levels, i.e., flood control (2 indicators), sediment discharge and siltation reduction (2 indicators) and power generation (1 indicator, i.e. cascade power generation upstream of Toudaoguai), under the current operation scheme than that under the pre-Long-Liu operation scheme, in which power generation attains a low significance. In the whole water–sediment–electricity system, the incoming sediment load into the Yellow River upstream of Toudaoguai and the entropy weight of the sediment inflow-outflow difference in the Ningxia-Inner Mongolia Reach frequently ranked among the top three indicators, accounting for more than 40% and up to 58%, respectively, of the total contribution under the current operation scheme, which are the key indices affecting system development. In future operation designs, special attention should be paid to the influences of operation on sedimentation in the Ningxia-Inner Mongolia Reach. Improving the coordination degree of water and sediment and reducing sedimentation in the Ningxia-Inner Mongolia Reach through certain methods, such as artificial flood process shaping by increasing the outflow of the Liujiaxia Reservoir to flush the channel before the summer-autumn flood season, could increase the operation efficiency and improve the development quality of the water–sediment–electricity system.

**Figure 5:**
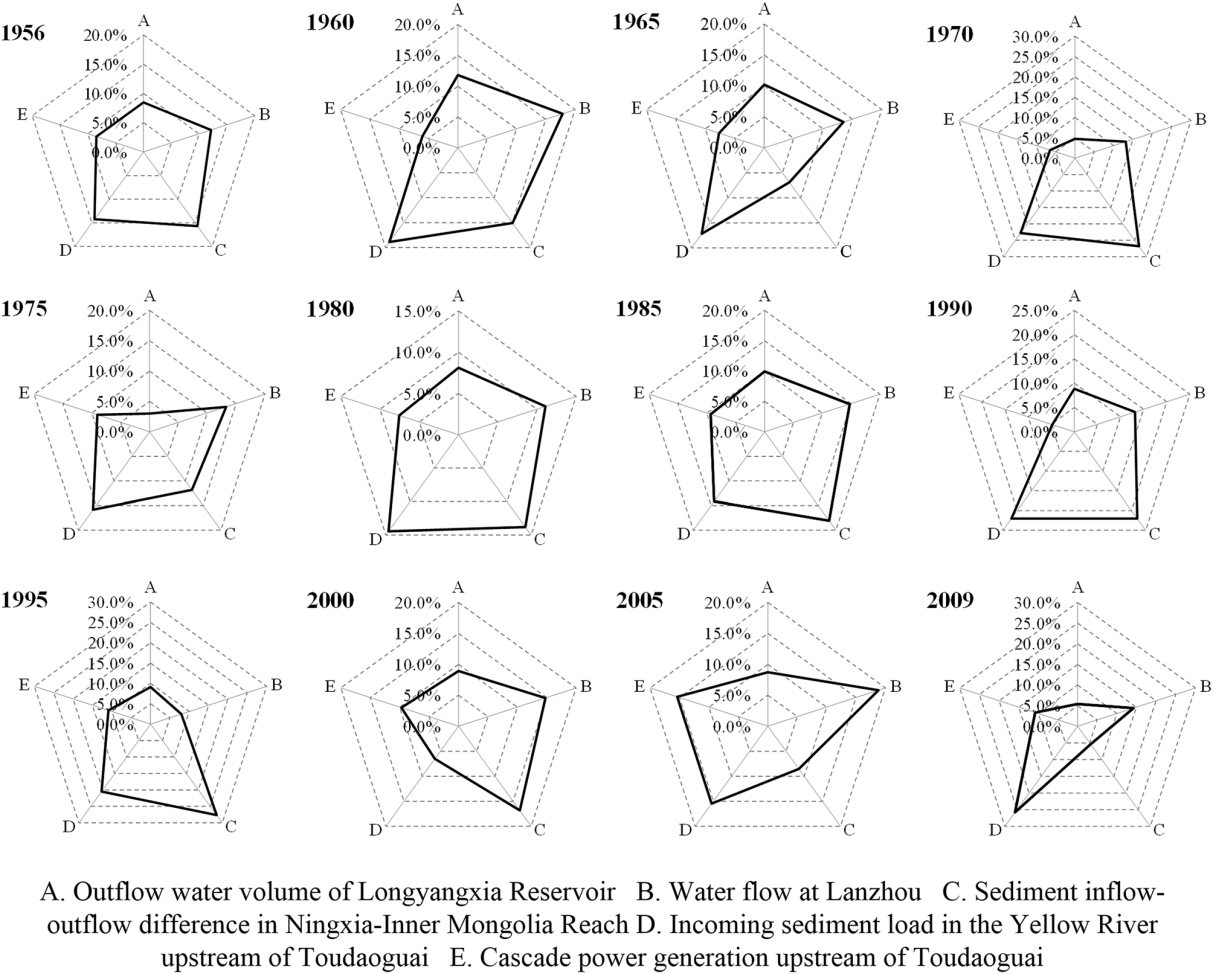
Entropy weight analysis of the key indices of the internal water–sediment–electricity system at the interannual scale in the current situation.

The variation characteristics of the contribution degree of the key indicators at the different time scales were analyzed. At the interannual scale, a time interval of 5 years was chosen to conduct the analysis. The high-flow years (1975, 1985, and 2005–2009) and low-flow years (1956, 1965, 1970, 1990–2000) were identified according to the inflow water volume of the Longyangxia Reservoir. Generally, during the relatively low-flow periods, the incoming sediment load into the Yellow River upstream of Toudaoguai and the sediment inflow-outflow difference in the Ningxia-Inner Mongolia Reach exerted greater impacts on the system, while during relatively wet periods with greater inflow, the water flow in the Lanzhou Reach exerted a greater impact on the system partly because of the higher flood control pressure faced by the lower reaches (Fig. 5).

**Figure 6:**
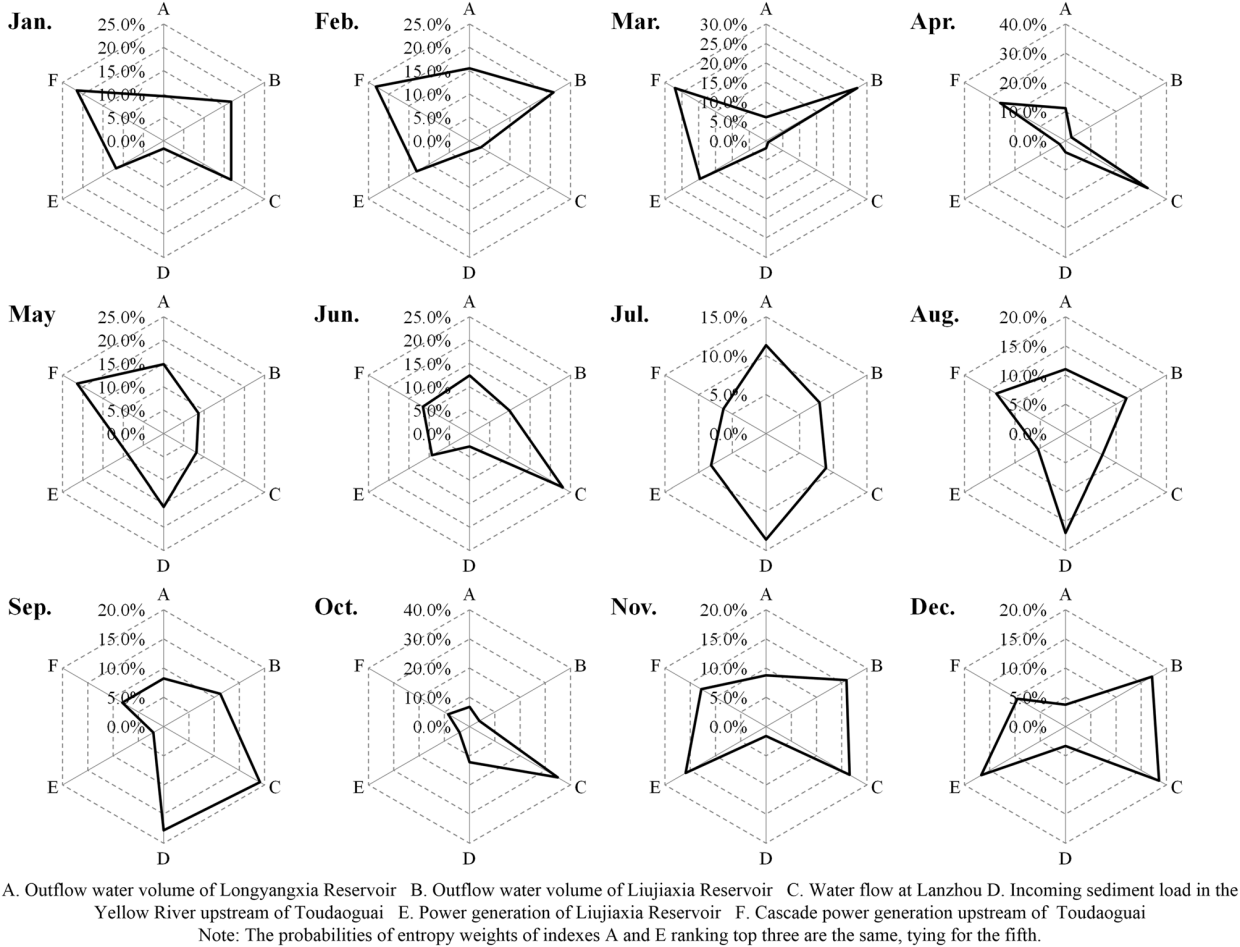
Entropy weight analysis of the key indices of the internal water–sediment–electricity system at the monthly scale in the current situation (The time period is 1956–2009).

During a year, water and sediment may vary in amount seasonally, and thus power generation. Therefore, the key indicators affecting the water–sediment–electricity system in each month were further analyzed based on monthly data from 1956 to 2009. At the month scale, the indicators greatly contributing to the system varied throughout the year with a certain periodicity (Fig. 6). From October to May of the following year, cascade power generation upstream of Toudaoguai yielded a greater contribution to the system, especially during the nonflood season. This mainly occurred because the joint Long-Liu operation scheme plays a vital role in the safe and stable operation of the Northwest Power Grid and improving the total power generation efficiency of cascade hydropower stations through both compensating and regulating roles. During the ice flood season, it is necessary to control the water flow in river channels, appropriately increase the subglacial discharge capacity of the river channels, prevent blocking of the river channels and ensure gradual freezing of the river channels under appropriate and steady flow conditions. In joint ice prevention regulation of the Longyangxia and Liujiaxia Reservoirs, the Liujiaxia Reservoir is expected to control the discharge flow according to the characteristics of ice running, river freezing and ice breaking. Therefore, the outflow of the Liujiaxia Reservoir is the key factor during this period. The contribution rate of the water flow at Lanzhou to the system is higher from April to June and from July to October. During these two periods, the joint Long-Liu operation scheme must satisfy the water consumption demand mainly via irrigation and flood control in cities along the Yellow River such as Lanzhou^8,9^. In addition, the incoming sediment load into the Yellow River upstream of Toudaoguai during the flood season is a key factor influencing the system.

### Study on coordinated development of the water–sediment–electricity system

**Figure 7:**
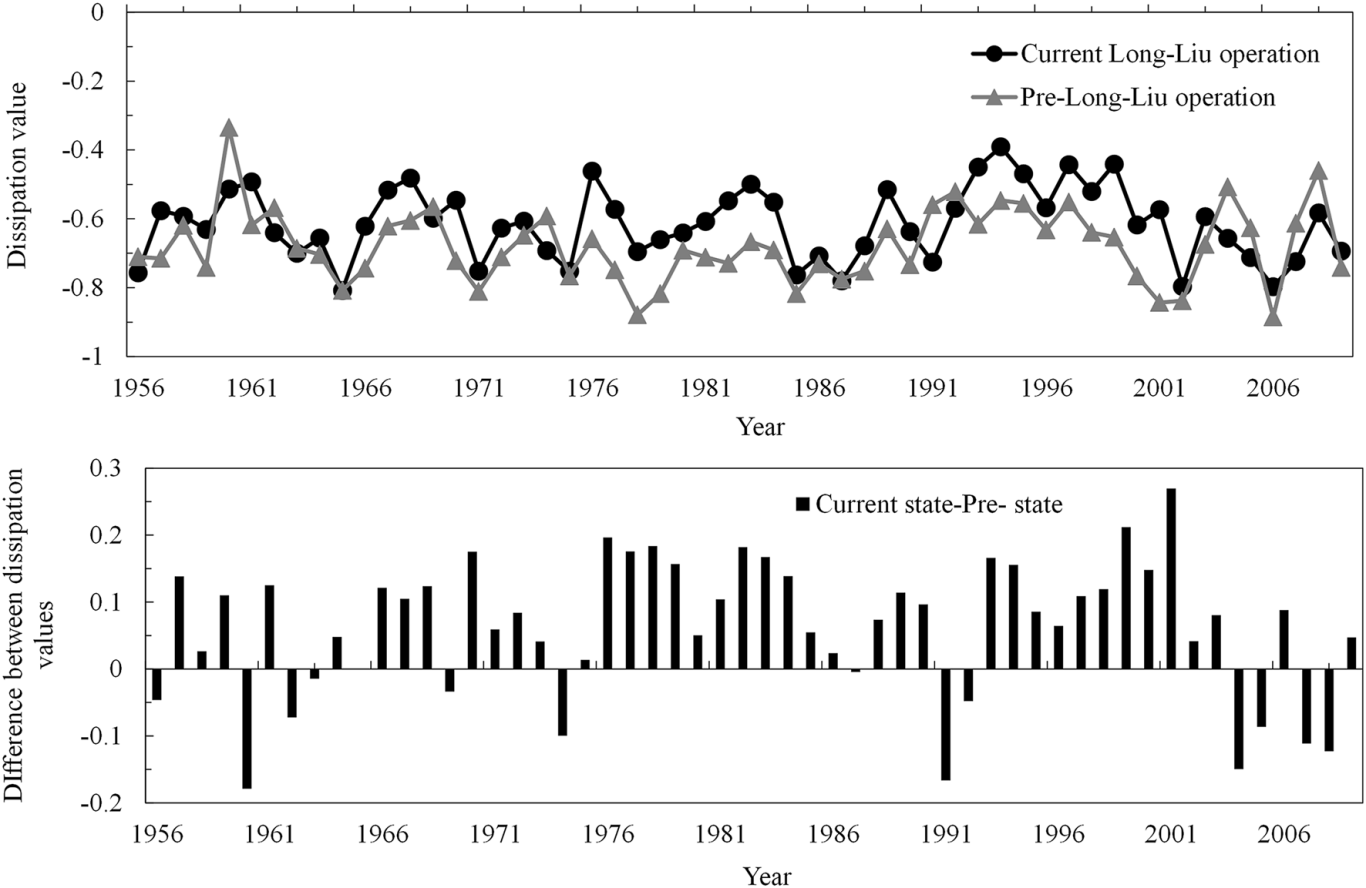
Comparison of the joint development level of the water–sediment–electricity system in the current situation and pre-Long-Liu operation state.

According to Fig. 7, the dissipation values of the water–sediment–electricity integrated system in both the current and the pre-Long-Liu operation states are lower than 0 from 1956 to 2009, indicating that coordinated development of the system is still at lower activity level. However, the dissipation value of the system in the current situation is generally higher than that in the pre-Long-Liu operation state, indicating that the coordination level of the water–sediment–electricity system under the current Long-Liu operation scheme is higher than that in the pre-Long-Liu operation state (Fig. 7). After 2003, under the current operation mode, the dissipation value decreases, which may result in sedimentation in the Liujiaxia Reservoir to a certain extent, and the reservoir may not be able to reduce the design dead water level as required for operation. According to the sedimentation degree, the minimum reservoir operating water level under appropriate water regulation is increased. The active storage capacity decreases, and the regulation capacity is reduced. Thus, normal reservoir operation is affected. In addition, overall, the dissipation value in the current situation remains stable with no obvious increase or decrease trend at the interannual scale, indicating that the current Long-Liu operation mode can only maintain a lower coordination level of the water–sediment–electricity system. Hence, it is necessary to gradually adjust and improve the joint operation mode of the upstream reservoir group, mainly including the Longyangxia and Liujiaxia Reservoirs, to enhance the coordination degree of water and sediment and the power generation level, thus enabling a higher-quality development of the water–sediment–electricity system and achieving high-level coordinated development of water, sediment and electricity.

## Discussions

The indices of the incoming sediment load into the Yellow River upstream of Toudaoguai and the sediment inflow-outflow difference in the Ningxia-Inner Mongolia Reach reflect the sediment discharge and siltation reduction effects of the reservoir operation scheme on the water–sediment–electricity coupling system. In the Ningxia-Inner Mongolia Reach, variations of the medium water channel, referring to the channel that could maintain the basic functions of flood relief and sediment transfer, would affect benefits of reservoir operation. Negative values of the index of the sediment inflow-outflow difference in the Ningxia-Inner Mongolia Reach suggest that sediment is removed from the channel, i.e., sediment discharge occurs. This could enlarge the channel, thus benefiting sediment transfer and flood relief. In contrast, positive values of the index of sediment inflow-outflow difference in the Ningxia-Inner Mongolia Reach suggest that more sediment is retained in the channel, i.e., sediment siltation occurs. This would reduce the channel volume, thus weakening the function of the medium water channel and increasing the risks of flooding and siltation to a certain extent. At the interannual scale, these key indices restrict the development quality of the water–sediment–electricity coupling system. The positive entropy exhibits an increase trend after 1993, indicating that the current operation mode of the Longyangxia and Liujiaxia Reservoirs does not resolve problems such as continuous siltation in the Ningxia-Inner Mongolia Reach. An uncoordinated relationship between water and sediment in the upper reaches is one of the key reasons leading to siltation in the Ningxia-Inner Mongolia Reach. To address this problem, on the one hand, it is necessary to improve the Long-Liu operation scheme, while on the other hand, it is necessary to build key hydropower complex projects in the main stream to further enhance the water and sediment regulation system of the Yellow River. The Heishanxia Reach is located downstream of Liujiaxia at the junction of Gansu and Ningxia in the upper section of the几 shaped river bend, which is closer to the Ningxia-Inner Mongolia Reach than the Longyangxia and Liujiaxia reservoirs. It is of great significance to curb the formation of a new suspended river in the Ningxia-Inner Mongolia Reach^31,32^. In the future, it is necessary to promote the establishment and construction of the Heishanxia High Dam as soon as possible. Re-regulation of the Heishanxia Reservoir should be applied to increase the sediment-transport water volume during the flood season and to retain sediment and reduce siltation, which can be achieved by shaping the flow processes of water and sediment to the benefit of the water and sediment transport process in the Ningxia-Inner Mongolia Reach, improving the relationship between water and sediment in the Ningxia-Inner Mongolia Reach, and restoring and maintaining the flood discharge capacity of the main river channel^31,32^, thus enhancing the quality of the water–sediment–electricity coupling system and the comprehensive benefits of joint reservoir operation.

Cascade power generation upstream of Toudaoguai is an important factor affecting the water–sediment–electricity coupling system, especially during the months with a lower inflow water volume. At present, there is still room to promote cascade power generation upstream of Toudaoguai. The regulation capacity of the Longyangxia and Liujiaxia reservoirs at the different time scales, from the daily scale to the multiannual scale, should be fully utilized to regulate runoff in the upper reaches of the Yellow River. Increasing the flow volume in the dry season could facilitate an increase in power generation, which could help to improve the guaranteed output and power generation of cascade hydropower stations, thus enhancing the system development quality.

During the water-consuming peak period (April-June), the entropy value is high under the current Long-Liu operation scheme, suggesting much room for optimization to reduce the operation uncertainty. During this period, the water flow in the Lanzhou Reach, indirectly reflecting the available water amount in the channel of the Ningxia-Inner Mongolia Reach, is a sensitive factor affecting the water–sediment–electricity coupling system. From April to June, spring irrigation, especially in the irrigation districts in the Ningxia and Hetao plains, is the most important consumer of water resources, which is generally sourced from the Yellow River. Therefore, from the perspective of reservoir operation, the storage capacity of the Longyangxia and Liujiaxia reservoirs should be fully utilized to regulate the outflow. Consequently, the seasonal distribution of runoff in the channel of the lower reach could be adjusted in accordance with the water utilization process, thus promoting the water guaranteed rate the Gansu, Ningxia and Inner Mongolia areas. In contrast, the water resources utilization rate during the spring irrigation period could be promoted through water-saving agricultural methods, such as sprinkler and drip irrigation methods, to reduce water consumption in the Ningxia-Inner Mongolia districts.

According to the above dissipation analysis, the dissipation value in the current state is lower than zero, indicating that the overall coordinated system development still occurs at a lower activity level. Generally, from the perspective of sedimentation reduction in river channels, water could be released to reasonable shape flood processes to flush the channel through joint reservoir operations. This, to a certain extent, requires an uneven runoff distribution throughout the year. In terms of water resources, the runoff distribution should be as uniform as possible to satisfy the continuous water demand in the lower reaches. This may be achieved by storing water during the flood season and replenishing the water demand during the dry period through proper water regulation. In contrast, from the perspective of power generation, maintenance of a high operation water head is more beneficial. Contradictions exist among the operation demands of water, sediment and power generation, and tradeoffs should necessarily be considered during joint operation scheme design. In future operation design, sensitive indicators during different periods, as suggested by radar charts (Figs. 5, 6), such as the sediment inflow-outflow difference in the Ningxia-Inner Mongolia Reach during the dry period, should be prioritized during tradeoff determination. In addition, a more flexible operation framework should be constructed through optimization algorithm improvement. According to certain factors, such as the siltation situation in river channels, inflow process and water demand, the outflow process of the Longyangxia and Liujiaxia reservoirs should be adjusted in a timely manner.

## Conclusions

This study applied systems thinking to quantitatively investigate the reliability of operation schemes and the development state of the water–sediment–electricity coupling system. At the interannual scale, the entropy reduction effect on the water–sediment–electricity coupling system under the current operation scheme is more notable, resulting in a higher reliability under the current operation scheme than that in the pre-Long-Liu operation state. During the summer-autumn flood season and ice flood season in a given year, the current operation scheme achieves a higher reliability. The contribution degrees of the various indicators to both the internal and external systems vary over time. At the interannual scale, the outflow water volume of the Liujiaxia Reservoir contributes more to the water–sediment–electricity system than do the other external factors in the current state. The key internal factors affecting system development during the low- and high-flow years include the incoming sediment load into the Yellow River at Toudaoguai, sediment inflow-outflow difference in the Ningxia-Inner Mongolia Reach and water flow at Lanzhou, respectively. Throughout the year, the internal indices affecting the development of the system originate from the different criterion levels and exhibit a certain periodicity. During the different periods, the key factors impacting the system vary. The outflow water volume of the Liujiaxia Reservoir yields a greater effect during the ice flood season. The water flow at Lanzhou imposes a greater effect during the spring irrigation season. The water flow at Lanzhou and the incoming sediment load into the Yellow River upstream of Toudaoguai are the key factors during the autumn flood season. Cascade power generation upstream of Toudaoguai exerts a greater effect during periods with little rainfall throughout the year. From 1956 to 2009, the system coordination level in the current state was higher than that in the pre-Long-Liu operation state, but the dissipation values of the water–sediment–electricity system were all lower than 0, indicating that the overall coordinated system development still occurs at a lower activity level.

In future joint operation scheme designs, the key indicators identified in this study should be prioritized, which could improve the development quality of the system more effectively. This can provide a basis for the implementation of precise policies in the reservoir operation process. In addition, the framework proposed based on systems thinking considering the coordination degree of the water–sediment–electricity system provides a new idea to evaluate and optimize operation schemes from the perspectives of river health and the socioeconomical demand.

## Data Availability

The data that supports the findings of this study are available upon request from the corresponding author. The data are not publicly available due to privacy or ethical restrictions.
